# Observations on the Declining Birth-Rate
*Part of a Thesis for the Degree of M.D.


**Published:** 1906-09-22

**Authors:** Thos. Divine

**Affiliations:** Late Assistant Medical Officer of Health County Borough of Huddersfield.


### HOSPITAL CLINICS.

### OBSERVATIONS ON THE DECLINING BIRTH-RATE.*

By THOS. DIVINE, M.D., C.M. (Glasg.), D.P.H.R.C.P.S. (Lond.), D.P.H. (Camb.),
Late Assistant Medical Officer of Health County Borough of Huddersfield.

### 1. THE BIRTH-RATE IN ENGLAND AND WALES.

DURING the year 1903, 948,271 births were registered, and the males were in proportion to the females as 1,035 is to 1,000. Calculated in proportion to total population at all ages, these births were equal to a rate of 28.4 per 1,000. The birth-rate in 1902 expressed in the same way was equal to 28.5, and the average birth-rate for the ten years, 1893-1902, was 29.4 per 1,000 persons living at all ages.

The birth-rate is found to vary widely in different parts of the country. Among registration counties, with populations exceeding 100,000, the birth-rate ranged in 1903 from 21.9 in Sussex, 22.6 in Cornwall, and 22.9 in Devonshire to 35.2 in Monmouthshire and in Glamorganshire, and 35.5 in Durham.[1]

Among the thirty-three large towns † in England and Wales the birth-rate in 1903 ranged from 21.1 in Halifax, 23.3 in Bradford, and 23.8 in Huddersfield to 33.7 in West Ham, 35.1 in Sunderland, and 35.8 in Gateshead.[2]

The following table shows the mean birth-rate of England and Wales, calculated per 1,000 total population at all ages, in three-year periods, embracing the four last census years, and on the estimated population in 1903 [3] :—

*Table I.*

| Three-year periods | | | | Year |
|---|---|---|---|---|
| 1870–72 | 1880–82 | 1890–92 | 1900–02 | 1903 |
| 35.3 | 34.0 | 30.7 | 28.6 | 28.4 |

It will be seen that the decline in the birth-rate from 1870-72 to 1903 is no less than 19.5 per cent.

To express birth-rates, however, in terms of total population at all ages is open to the objection that it does not take account of the age constitution of the population, and, in particular, that it does not have regard to the female population at conceptive ages. The birth-rate, it may be taken, must needs be ruled by the number of women living aged from 15 to 45 years.

The importance of bearing this in mind in connection with birth-rates is brought out by a consideration of the following facts. The census returns from 1871 to 1901 show that both in 1871 and in 1881 the proportion of women aged 15 to 45 was 23.1 per cent. of the total population; in 1891 the proportion was 23.8 per cent., and had increased to 25 per cent. in 1901. On the other hand, the returns from 1871 to 1901 show that the female population aged 15 to 45 contained not an increasing but a constantly decreasing proportion of married women; and, further, that among these married women, the proportion of those at ages under 25 years has continuously decreased. Thus, of the total number of married women, (15 to 45 years), the proportion of those aged 15 to 25 years was 15.2 per cent. in 1871, 14.8 per cent. in 1881, 13.7 per cent. in 1891, and as low as 12.4 per cent. in 1901.

It therefore becomes evident that a more accurate method by which to express the birth-rate would be to take account, as far as possible, of these varying factors. But the age and marital condition of the female population is only known with accuracy at or about census periods. At these periods, however, three other methods of expressing the birth-rate become possible. It may be expressed as: (1) The proportion of total births per 1,000 women aged 15 to 45 years; (2) the proportion of legitimate births per 1,000 married women aged 15 to 45 years; (3) the proportion of illegitimate births per 1,000 unmarried and widowed women aged 15 to 45 years.

The following table gives the birth-rates as calculated by these three alternative methods in three-year periods, embracing the four last census years, and on the estimated population of each class in the year 1903. The percentage of decline of the birth-rate from the three-year period 1870-72 to the year 1903 is also set forth.[4]

*Table II.*

| Aged 15 to 45 years | Birth-rate per 1,000 | | | | | Percentag decline 1870–72 to 1903 |
|---|---|---|---|---|---|---|
| | 1870–72 | 1880–82 | 1890–92 | 1900–02 | 1903 | |
| All women ... | 153·7 | 147·7 | 129·7 | 114·8 | 113·8 | 26·0 |
| Married women | 292·5 | 286·0 | 263·8 | 235·5 | 233·3 | 20·3 |
| Unmarried and Widowed ... | 17·0 | 14·1 | 10·5 | 8·5 | 8·4 | 50·9 |

Calculated on the total population at all ages the decline per cent. from 1870-72 to 1903 was shown to be 19.5; but the calculations by the more accurate methods, as have been done in the above table, prove that the true decrease which has occurred is not adequately shown by simply calculating the rate on the total population; and that it therefore fails to disclose the amount of decrease that has actually occurred. In the Sixty-sixth Annual Report of the Registrar-General it is pointed out that if the average fecundity of the female population at ages 15 to 45 had remained constant, the births in proportion to the total population would have increased during the past 30 years by nearly 2 per cent. Stated in another way, this means that had the ratio

* Part of a Thesis for the Degree of M.D.

† The 33 large towns referred to here are: London, Croydon, West Ham, Brighton, Portsmouth, Norwich, Plymouth, Bristol, Wolverhampton, Birmingham, Leicester, Nottingham, Liverpool, Manchester, Salford, Oldham, Bradford, Leeds, Sheffield, Hull, Sunderland, Gateshead, Newcastle-on-Tyne, Derby, Birkenhead, Bolton, Blackburn, Burnley, Preston, Huddersfield, Halifax, Cardiff, and Swansea.

of births to the female population of conceptive ages been identical in 1871 and in 1903, the births registered in the latter year would have amounted to upwards of one and a quarter millions, instead of 948,271 actually recorded.

### 2. Illegitimate Birth-Rate.

During the last fifty years the number of illegitimate births per 1,000 population at all ages has varied from a maximum of 2.3 in the years 1859, 1863, and 1864 to a minimum of 1.1 in each of the years 1900 to 1903. In the latter year 37,302 illegitimate births were registered.

The illegitimate birth-rate has declined with the legitimate birth-rate, and the true extent to which this has occurred since 1870-72 is shown in Table II., where the calculation is made as a proportion to unmarried and widowed women aged 15 to 45 years.

The decline in the illegitimate birth-rate, though it contributes to a small extent to the decline in the general birth-rate, cannot, of course, be regarded as other than satisfactory, not only on grounds of morality, but also on account of the important bearing which illegitimacy has on infantile mortality, the mortality among illegitimate infants being, as a rule, about double that of infants born in wedlock.

### 3. Still-Births.

The consideration of this subject is closely connected with that of the declining birth-rate. Neech,[5] from inquiries in Halifax over a series of years, gives data which show that out of every 1,000 births 50 are stillborn; while Greenwood found that in Blackburn,[6] in the year 1904, 64 still-births occurred in 1,000 births. No provision exists in England for the registration of still-births. There can be no question that this is a condition of affairs calling for remedy. The report of the Committee of the House of Commons on Death Certification [7] says: "There is reason to think that if the statistics on the subject could be obtained, it would be found that the number of children buried in the United Kingdom annually as stillborn is enormous," and the Committee are further convinced that "the absence of legal requirement that such still-births should be registered prior to disposal of the bodies is fraught with very serious danger to child life." That this danger is especially great in the case of illegitimate children can readily be understood. One of the recommendations of the Inter-Departmental Committee on Physical Deterioration is that still-births should be registered.[8] The statutory rules under the Midwives Act of 1902, which came into force on April 1, 1905, made a step in the right direction by requiring midwives to immediately give notice to the authorities whenever a still-birth occurs in their practice. If all still-births were required to be officially certified and registered, it would be an easy matter to institute inquiries where the circumstances seemed to demand them; and this would be one means of checking the grave irregularities which undoubtedly exist.

### 4. The Birth-Rate Declining.

A reference to the Annual Reports of the Registrar-General shows that during recent years there has been a marked decline in the birth-rate, not only in England and Wales, but also in most British Colonies and other large civilised countries of the world. The extent to which this has occurred in England and Wales, and the manner of the decline, will be seen by a reference to Tables I. and II.

If the birth-rate equals the death-rate, and immigration balances emigration, it is obvious that no numerical change occurs in a population. The increase or decrease of any population is governed therefore by two factors: (1) The difference between the birth-rate and the death-rate; (2) the difference between immigration and emigration.

If the birth-rate exceeds the death-rate in a stationary population, the excess of births over deaths gives the *natural* increase; and, conversely, if the death-rate exceeds the birth-rate, the excess gives the *natural* decrease of that population. In the same way, the difference between immigration and emigration produces a further change in one direction or the other.

The net result between these two factors determines the actual (or total) increase or decrease.

An examination of the report on the census of 1901 [9] discloses the fact that, although the actual increase (12.16 per cent.) during 1891-1901 in England and Wales was at a higher rate than that during 1881-1891, when the rate of increase was 11.66 per cent., the growth in the population was not due to any greater rate of natural increase, but is actually associated with a decline in that rate. In other words, the higher rate of actual increase in the decade 1891-1901 is not due to a higher rate of natural increase, but to a decline in emigration or to an increase in immigration. The natural increase as determined by the excess of births over deaths, and also the actual increase as finally determined by the other factor in England and Wales, from 1871, is shown in the following table:—

*Table III.*

| Period | Natural increase per cent. | Actual increase per cent. |
|---|---|---|
| 1871-1881 ... ... | 15.09 | 14.36 |
| 1881-1891 ... ... | 13.97 | 11.66 |
| 1891-1901 ... ... | 12.39 | 12.16 |

This result is due, for the most part, to the continuous decline in the birth-rate which has characterised these periods, and is arrived at in spite of the simultaneous decline in the general death-rate which has occurred.

The following table [10] shows the average birth-rate for twenty-four different countries and British Colonies arranged in the order of highest birth-rate. For convenience the average infantile mortality is also here set forth.

Of these, the birth-rate in Ireland in 1903 was the same as the average for the decade 1893-1902. In four British Colonies—namely, Ceylon, Jamaica, Western Australia, and New Zealand—the rates in 1903 exceeded the average. The returns for the German Empire and for Tasmania are not to hand for the year 1903. Of the remaining seventeen countries shown in the table, the birth-rate in 1903 was below the average.

*Table IV.—Average Birth-Rates and Infantile Mortality for ten years,* 1893-1902.

| Country | Births per 1,000 living | Deaths of children under one year, per 1,000 births | Country | Births per 1,000 living | Deaths of children under one year, per 1,000 births |
|---|---|---|---|---|---|
| Russia (European) ... ... | 48.6 (1) | 272 (1) | Tasmania ... | 29.6 | 93 |
| Hungary... ... | 40 0 | 224 | New South Wales... ... | 29.1 | 111 |
| Jamaica ... ... | 39.1 | 171 | Belgium ... | 28.9 | 157 |
| Prussia ... ... | 36.5 | 199 | Western Australia ... ... | 28.9 | 146 |
| German Empire. | 35.9 | 195 (2) | Switzerland ... | 28.3 | 145 |
| Ceylon ... ... | 35.6 | 170 | South Australia | 27.9 | 146 |
| Chili ... ... | 35.6 | 333 | Victoria ... | 27.1 | 109 |
| Italy ... ... | 34.3 | 173 | Sweden ... ... | 27.0 | 99 |
| The Netherlands | 32.3 | 152 | New Zealand ... | 26.3 | 81 |
| Norway ... .... | 30.2 | 94 | Ireland ... ... | 23·1 | 104 |
| Denmark... ... | 30.0 | 133 | France ... ... | 22.0 | 158 |
| Queensland ... | 30.0 | 103 | | | |
| Scotland ... ... | 29.9 | 127 | | | |

(1) For ten years, 1890–1899. (2) For two years, 1901-1902.

Special interest attaches to the returns from New South Wales, where the birth-rate had declined from the average of ten years, 29.1 to 25.3 in the year 1903.[11] The New South Wales Government appointed a Royal Commission in 1903 to inquire into the decline in the birth-rate in that colony. The following table [12] from the report of this Commission indicates the manner in which the decline in the birth-rate of the several colonies has come about. The birth-rates per 1,000 population at all ages as shown in successive quinquennia from 1871 to 1900. I have added the rates for the census year 1901.[13]

*Table V.*

| Period | New South Wales | Victoria | Queensland | South Australia | Western Australia | Tasmania | New Zealand |
|---|---|---|---|---|---|---|---|
| 1871–1875... | 39.05 | 35.69 | 40.81 | 37.24 | 31.30 | 29.72 | 40.02 |
| 1876–1880... | 38.53 | 31.43 | 36.72 | 38.28 | 32.97 | 31.54 | 41.32 |
| 1881–1885... | 37.65 | 30.76 | 36.37 | 38.52 | 34.57 | 35.02 | 36.50 |
| 1886–1890... | 36.36 | 32.72 | 38.81 | 34.48 | 36.88 | 34.59 | 31.22 |
| 1891–1895.... | 32.93 | 30.93 | 35.15 | 31.54 | 30.77 | 32.84 | 27.66 |
| 1896–1900... | 27.98 | 26.22 | 30.40 | 26.59 | 28.73 | 28.28 | 25.74 |
| 1901 ... | 27.6 | 25.7 | 28.3 | 25.4 | 30.4 | 28.4 | 26.3 |

The decline has been least marked in Western Australia and Tasmania, but in every other colony it has been considerable. The figures for New South Wales are very striking, a decline from over 39 to less than 28 having been arrived at in marked and successive down-steps, unrelieved by even a pause.

Statistics from Canada leave little doubt that the birth-rate among the inhabitants of British origin is remarkably low. In Ontario, where British stock predominates, the birth-rate in 1901 was only 21.1; in the province of Quebec, which is chiefly French Canadian, it was 35. In the year 1902 the mean birth-rate of the thirty-five counties of which the province of Quebec is comprised was 42.2, reaching no less than 53.2 in the county of Beauce.[14] In the city of Montreal the population is divided into three classes—namely, French Canadian, other Catholics, and Protestants. The birth-rate in 1902 for each class was as follows [15]:—

*Table VI.*

| Three classes | Birth-rate per 1,000 population |
|---|---|
| French Canadian Catholics ... ... | 43.5 |
| Other Catholics ... ... ... | 22.4 |
| Protestants ... ... ... ... | 23.7 |

The high birth-rate of the "French Canadian Catholics," who are orthodox Roman Catholics, is remarkable. It should also be noted how closely the rate for "Other Catholics," who are not orthodox Roman Catholics, approaches to that of the average for France, 22.0. Newsholme draws an important conclusion from facts like these, which will presently be noted.

### 5. Causes of the Decline in the Birth-Rate in England and Wales.

It is pertinent to inquire, What are the causes of this decline in the birth-rate?

For the purpose of statistics the child-bearing age may be considered as from 15 to 45 years. Therefore the age of women at marriage is the chief physiological factor determining the proportion of children to a marriage. The age of women at marriage has been steadily rising in this country for over a quarter of a century. In the quinquennium 1876-1880 the proportion of minors among wives was 217 per 1,000 marriages, and the proportion has successively declined from that period to 152 in the year 1903. The manner of the change is shown in the following table [16]:—

*Table VII.*

| Period. | Women under age per 1,000 marriages. | Period. | Women under age per 1,000 marriages. |
|---|---|---|---|
| 1876–1880 ... | 217.0 | 1896–1900 ... | 168.0 |
| 1881–1885 ... | 215.0 | 1901 ... ... | 159.9 |
| 1886–1890 ... | 200.2 | 1902 ... ... | 153.7 |
| 1891–1895 ... | 182.6 | 1903 ... ... | 152.3 |

No reliable statistics exist in this country showing the age of mothers at the birth of their children; and so the fecundity of women at different ages cannot be determined. M. Körösi,[17] however, has shown that the fecundity of the female in Budapest reaches its maximum between the eighteenth and nineteenth year, descending then in a regular line to the age of 45 to 50, when it ceases. Every hundred marriages of girls under 18 years of age only produce within a year 36 to 38 infants. From 18 to 20 years fecundity reaches its maximum of 40 per cent.—*i.e.* 40 children within a year. At 25 years it is 32 per cent.; at 30 years it is 24 per cent.; at 35 years 17 per cent.; at 40 years scarcely 7 per cent.; at 50 years 0.1 per cent.

It is significant to note that the decline in the birth-rate in England and Wales is coincident with a change in the age constitution of married women; and, from the researches of Körösi in Budapest, it seems probable that this change in the age constitution of married women and the decline in the birth-rate in England and Wales may stand in some measure in the relation of cause and effect.

The marriage-rate in England and Wales has been declining through three decades, though this is not obvious from the marriage-rate as usually stated—that is, persons married per 1,000 living at all ages. Thus, the mean marriage-rates [18] in the three year periods (containing in each period the census year) 1880-1882, 1890-1892, 1900-1902 were respectively equal to 15.2, 15.5, and 15.9 persons married per 1,000 total population, and would suggest a steady rise in the marriage-rate. But, since a large majority of the population are either already married, or are below the minimum age at which marriages take place, the total population is not a satisfactory standard by which to measure the rate of marriage. If the rates be calculated on the *unmarried and widowed portion* of the population at ages *above* 15 years, the apparent increase is turned into a decrease thus:—

*Table VIII.*

| Three year periods | Persons married per 1,000, unmarried and widowed, above 15 years of age |
|---|---|
| 1880-1882 ... ... ... | 51.5 |
| 1890-1892 ... ... ... | 49.8 |
| 1900-1902 ... ... ... | 48.7 |

The marriage-rate in England and Wales has, therefore, decreased in proportion to that section of the population among which marriages take place. This must in some degree contribute to the decline in the English birth-rate.

As is well known, France, in regard to a falling birth-rate, is, and has for a long time been, in a much worse position than this country. One writer [19] (Emile Macquart) looks on the existing condition with Gallic complacency, and suggests that civilisation and a low birth-rate go hand in hand; and that in this respect, as in others, France is simply in the van of civilisation. Arseni Dumont,[20] on the other hand, looks at the subject more seriously, and, I would say, more sensibly. He finds that the age at marriage is steadily rising. Of the whole male population of France aged 15 to 24 years, 3.6 per cent. are married; of those aged 20 to 24, 7 per cent. are married; and at ages 25 to 29 years, 50 per cent. are married. Again, amongst males from 18 to 49 years of age, 45.8 per cent. are unmarried, and among females between 15 and 49 years, 44.9 per cent. are single. Why do not these two unmarried groups come together? Dumont enumerates several reasons: (1) State of society—increasing difficulty for young men to establish themselves in life; (2) dotal difficulty; (3) military service; (4) celibate example of Church; (5) tendency of the age to personal comfort.

There is reason to believe that drunkenness is on the increase among women. Several competent witnesses before the Inter-Departmental Committee on Physical Deterioration testified to this in no uncertain manner. The relation of "cirrhosis of the liver" to intemperance is well recognised. The following table [21] gives the average male and female death-rate per million living from this cause in two quinquennial periods twenty-five years apart:—

*Table IX.*

| Average | Male death-rate | Female death-rate |
|---|---|---|
| 1873–77 ... ... | 111 | 74 |
| 1898–1902 ... ... | 155 | 115 |
| Difference ... | +40% | +55% |

It is well known that alcoholism is a cause of sterility, of still-births, and of premature births. At any rate the infantile death-rates from premature birth and from congenital defects have enormously increased during recent years; and it has been shown that about 50 still-births occur for every 1,000 infants born alive. Alcoholism therefore probably plays a not unimportant part in the decline in the birth-rate. Moreover, there are many other ways in which the baneful influence of alcohol (in either sex) may indirectly operate in the same direction.

So far as the Registrar-General's returns [22] can show there is no reason to believe that syphilis is more operative now than formerly, both the male and female death-rates from syphilis having irregularly declined from 1884 to 1902. In 1903, however, there was a slight rise in the death-rate from syphilis for both sexes. The statistics for syphilis, however, are notoriously unreliable.

It has already been noted that the illegitimate birth-rate in England and Wales has declined about 50 per cent.; and this must in a small measure contribute to the decline in the general rate.

It would appear that the decline in the birth-rate is not equally shared by all classes of the community. Slumdom still makes a liberal contribution to the population of our great cities. Moreover, it will be shown that this is the class of community in which infantile mortality is highest. Bertillon,[23] at a meeting of the International Statistical Institute at St. Petersburg, September 1897, gave the following statistics as to the births per 1,000 women aged 15 to 50 per annum in different quarters of the under-noted cities:—

*Table X.*

| Classification | Paris | Berlin | Vienna | London |
|---|---|---|---|---|
| Very poor quarters ... | 108 | 157 | 200 | 147 |
| Poor quarters ... ... | 95 | 129 | 164 | 140 |
| Comfortable quarters ... | 72 | 114 | 155 | 107 |
| Very comfortable quarters | 65 | 96 | 153 | 107 |
| Rich quarters ... ... | 53 | 63 | 107 | 87 |
| Very rich quarters ... | 34 | 47 | 71 | 63 |
| Average ... ... | 80 | 102 | 153 | 109 |

If the marriage- and birth-rates of certain registration districts in London, selected on social grounds, be calculated for the year 1903, on the census population of 1901, the following result is obtained.[24] The infant deaths per 1,000 births in 1903 are also shown:—

*Table XI.*

| Rate per 1,000 persons living | St. George's, Hanover Square | Kensington | Hackney | Stepney | Whitechapel |
|---|---|---|---|---|---|
| Marriage-rate ... | 21.3 | 18.4 | 14.8 | 15.1 | 14.4 |
| Birth-rate ... | 17.5 | 20.3 | 26.9 | 32.3 | 36.3 |
| Infantile mortality | 141 | 145 | 128 | 208 | 194 |

If other large towns be examined in the same way the same general tendency is found—namely, the lower the stratum of society, the higher is the birth-rate, and the higher also is the rate of infantile mortality.

It would seem as if those who can best afford to have large families, and bring them up respectably, have very small families, while the parents in the slums have, as a rule, very large ones; and since there is no reason to believe that social status *per se* has any effect upon fecundity, one is therefore forced to the conclusion that much of the decline in the birth-rate is due to deliberate restraint. Newsholme,[25] from an examination of certain factors, accepts the conclusion that it is quite clear that the main cause of the diminution in the birth-rate is "the deliberate and voluntary avoidance of child-bearing on the part of a steadily increasing number of married people, who not only prefer to have but few children, but who know how to obtain their wish." That this is the chief reason is shown by the extremely high birth-rate among the French population in Canada and the abnormally low birth-rate in France. The difference, as Newsholme observes, is inexplicable on the score of climate, or, indeed, on any other known cause, except that the former, who are orthodox Roman Catholics, are prohibited by their religious belief from practising the artificial means of preventing large families which finds favour in France. In further support of this I would point out that in the orthodox Roman Catholic country of Spain the birth-rate for the last quarter of a century has maintained a high level, being 35.8 in 1879 and 36.4 in 1903.[26]

It would therefore appear that the decline in the birth-rate in England and Wales is due to the following factors:—

1. Rise in the age of women at marriage.
2. Decline in the marriage-rate.
3. Increase of drunkenness among women leading to still-births and sterility.
4. Decline in illegitimacy.
5. Deliberate restraint: perhaps the most important factor.

[1] Sixty-sixth Annual Report Registrar-General. [2] Annual Summary of Registrar-General, 1903. [3] Sixty-sixth Annual Report Registrar-General. [4] Compiled from Sixty-sixth Annual Report Registrar-General. [5] Neech, "Annual Report on the Health of the County Borough of Halifax for 1903." [6] Greenwood, "Annual Report upon the Health of Blackburn for 1904." [7] "Report of the Select Committee of the House of Commons on Death Certification," Sept. 1893. [8] Report of the Inter-Departmental Committee on Physical Deterioration, 1904, p. 88. [9] General Report on the Census, England and Wales, 1901, p. 16. [10] Compiled from the International Vital Statistics, Sixty-sixth Annual Report Registrar-General, p. clxxvi. et seq. [11] International Vital Statistics Sixty-sixth Ann. Rep. Reg.-General. [12] Report of the Royal Commission on the Decline of the Birth-Rate and on the Mortality of Infants in New South Wales, 1904. [13] International Vital Statistics Sixty-sixth Annual Report Registrar-General. [14] "Annual Report of the Board of Health of the Province of Quebec," 1902-1903 (Quoted by McCleary: "Infant Milk Depots," p. 11). [15] "Annual Report of the Medical Officer of Health of the City of Montreal," 1902 (Quoted by McCleary: "Infant Milk Depots," p. 11). [16] Sixty-sixth Annual Report Registrar-General, p. xl. [17] Quoted by Newsholme: "Vital Statistics," 1899, p. 66. [18] Sixty-fifth Ann. Rep. Reg.-Gen., p. vi. [19] Bull. et Mem. Soc. d'Anthrop. de Paris, 5S. iii., 1902 (Noticed in B.M. Jour., 1903, vol. i., p. 267). [20] Ibid. [21] Sixty-fifth Ann. Rep. Reg.-General, p. lvi. [22] Sixty-sixth Ann. Rep. Reg.-General. [23] Newsholme's Vital Statistics, 1899, p. 75. [24] Calculated from data in Sixty-sixth Ann. Rep. Reg.-General, and in Registrar-General's Summary, 1903. [25] Newsholme: "Vital Statistics," 1899, p. 79. [26] International Vital Statistics: Sixty-sixth Annual Report, Registrar-General.

